# The Effect of Captivity on the Dynamics of Active Bacterial Communities Differs Between Two Deep-Sea Coral Species

**DOI:** 10.3389/fmicb.2018.02565

**Published:** 2018-10-29

**Authors:** Pierre E. Galand, Leila Chapron, Anne-Leila Meistertzheim, Erwan Peru, Franck Lartaud

**Affiliations:** ^1^Sorbonne Université, CNRS, Laboratoire d’Ecogéochimie des Environnements Benthiques (LECOB), Observatoire Océanologique de Banyuls, Banyuls-sur-Mer, France; ^2^Sorbonne Université, CNRS, Laboratoire d’Océanographie Microbienne (LOMIC), Banyuls-sur-Mer, France

**Keywords:** *Lophelia pertusa*, *Madrepora oculata*, DNA/RNA, bacteria, aquaria experiment

## Abstract

Microbes play a crucial role in sustaining the coral holobiont’s functions and in particular under the pressure of environmental stressors. The effect of a changing environment on coral health is now a major branch of research that relies heavily on aquarium experiments. However, the effect of captivity on the coral microbiome remains poorly known. Here we show that different cold-water corals species have different microbiome responses to captivity. For both the DNA and the RNA fraction, *Madrepora oculata* bacterial communities were maintained for at least 6 months of aquarium rearing, while *Lophelia pertusa* bacteria changed within a day. Interestingly, bacteria from the genus *Endozoicomonas*, a ubiquitous symbiont of numerous marine hosts, were resilient and remained active in *M. oculata* for several months. Our results demonstrate that a good knowledge of the coral microbiome and an understanding of the ecological strategy of the holobiont is needed before designing aquarium experiments.

## Introduction

Corals and microbes form together an holobiont that is built upon the complex and intricate relationship between the cnidarian host and its associated microbial community. The role of the microbiome in sustaining the coral holobiont’s function is still poorly known but it certainly plays an important role for the coral nutrition and health (Bourne et al., 2016). Corals health is particularly sensitive to environmental changes. Water temperature, quality, and acidification are, among others, factors that strongly impact the corals’ ecology and physiology, and that have been the focus of a number of studies. Recent works have emphasis the need to characterize the microbial communities associated to the host to better understand the impact of environmental conditions on corals (Kelly et al., 2014).

Studying the effects of environmental changes on corals often requires manipulating the corals surrounding environment. Such operations are often easier to achieve under controlled conditions in aquaria. Aquaria experiments require the maintenance of coral in captivity for acclimation during long periods. During that time, the aquarium conditions of rearing, although mimicking natural conditions, are always different from the ones found in nature. Nevertheless, captive corals can be kept alive and remain apparently healthy for long periods of time (Carlson, 1987; Borneman, 2008), including deep-sea corals that exhibit growth rates comparable to the *in situ* ones (Orejas et al., 2011; Form and Riebesell, 2012; Lartaud et al., 2014). However, as the importance of taking into account the entire holobiont in coral studies has emerged, the question remains as whether the coral associated microbes are strongly impacted by the captivity of the host. If the microbiome is indeed impacted, results obtained in aquaria should be extrapolated to the natural coral colonies with caution. To date, the few existing comparisons between *in situ* and aquaria coral microbiomes suggest that captivity always changes the coral’s bacterial communities (Kooperman et al., 2007; Schöttner et al., 2009; Pratte et al., 2015; Röthig et al., 2017). However, studies remain scarce and they often encompass a few samples only, analyzed with different protocols and gathered with diverse sampling strategies.

The importance of aquaria experiments for studying corals is critical for species that are hard to sample and monitor *in situ*. It’s the case for deep-sea corals, also named cold-water corals, which are in most cases found at depths not accessible by divers. Cold-water corals provide important ecosystem services by forming deep-sea reefs that represent hot spots of biodiversity in many regions of the world (Roberts et al., 2006). Among cold-water corals, *Lophelia pertusa* and *Madrepora oculata* are the two key species found globally. They often grow in the same sites, but they can show different responses to environmental changes such as temperature variations (Naumann et al., 2014). The physiological differences may be related to their distinct reproductive strategy (Waller and Tyler, 2005), feeding or assimilation/storage efficiencies (Kiriakoulakis et al., 2005) or association to different bacterial communities (Hansson et al., 2009; Schöttner et al., 2012; Meistertzheim et al., 2016). The different communities could reflect different ecological strategies with a resilient bacterial consortium associated to *M. oculata*, which fits the strict definition of holobiont, and a more versatility microbiome for *L. pertusa*, which suggests an association that is more influenced by environmental conditions or nutritional status (Meistertzheim et al., 2016).

The goal of this study was to test if the microbiomes associated to *L. pertusa* and *M. oculata* changed under captivity. We chose these two species as models representing the contrasted cases of an “unfaithful” versus a “loyal” microbiome. We maintained the two cold-water coral species in aquaria and monitored their associated bacterial communities at different time scales ranging from 1 day to 2 years. We targeted both the 16S ribosomal RNA genes (DNA) and the 16S ribosomal RNA (RNA) to compare the entire bacterial assemblage to the active fraction of the community (Galand et al., 2015).

## Materials and Methods

### Coral Sampling and Rearing

Corals were sampled in the Lacaze-Duthiers submarine canyon off the coast of Banyuls-sur-Mer in the northwestern Mediterranean Sea (42°32′0.72′′ N; 03°25′0.26′′ W) at ∼530 m depth. Healthy looking coral fragments were randomly collected from three colonies of *M. oculata* and three colonies of *L. pertusa* growing on a small coral reef area (2,400 m^2^) located at the base of the western flank of the canyon. The corals were collected sequentially using a Remotely Operated Vehicle (ROV) deployed from the R/V Minibex Vessel (COMEX SA). They were placed in separate polypropylene boxes that were closed *in situ* to maintain the ambient bottom water temperature (∼13°C) during the transport to the surface, and avoid cross contamination between samples. On board, the coral fragments meant as time 0 samples were flash frozen in liquid nitrogen and the rest of the coral fragments were transferred to aerated 30 L seawater tanks maintained in the dark at 13°C.

Once in the laboratory, live corals were fixed to cement blocks using an aquatic epoxy resin and positioned in a 80 L aquarium in the dark in a thermo-regulated room that received continuous flow (>1 renewal day^-1^) of filtered (5 μm) Mediterranean seawater pumped from 5 m depth. To mimic deep-sea temperature stability, the aquarium sea water was maintained year-round at 13°C ±0.5°C by controlling the room temperature and by using a storage tank coupled to a chiller for regulating the incoming sea water temperature. Corals were fed three times a week with freshly hatched *Artemia salina* nauplii (1000 *A. salina* L^-1^) (Lartaud et al., 2013).

Coral fragments were sampled after 1, 5, 20, 60, 180, 270, and 730 days of captivity. For *L. pertusa*, we had fewer fragments and did not sample at 180 days. Coral samples were flash frozen in liquid nitrogen and then stored at -80°C. In addition, 5 L of aquarium water were sampled at time 1, 5, 20, and 60 days, and filtered on 0.22-μm pore-size polycarbonate filters (Millipore) after prefiltration through 3-μm pore-size polycarbonate filters (Millipore). Filters were stored at -80°C until nucleic acid extraction.

### DNA and RNA Extractions and Sequencing

For each sampling time, DNA and RNA was extracted from three different polyps for each species. Polyps were crushed separately using a hammer and the tissues were homogenized in tubes containing a garnet matrix using a FastPrep Instrument (MP Biomedical, Santa Ana, CA, United States). The samples were then divided into two tubes, one for RNA extraction and one for DNA extraction. RNA and DNA were extracted using, respectively, the Maxwell^®^ simply RNA Tissues Kit LEV and the Maxwell^®^ Blood DNA Purification Kit LEV (Promega, Madison, WI, United States) on a Maxwell 16 MDx Instrument (Promega) following the manufacturer instructions. For the water bacteria communities, DNA and RNA were extracted from filters following our standard protocol (Galand et al., 2015). Briefly, cells were lysed with freshly prepared lysozyme solution applied directly to Sterivex cartridges, and a second incubation after adding proteinase K, followed by extraction using the AllPrep DNA/RNA kit (Qiagen). DNA and RNA concentrations were measured by spectrophotometry (Nanodrop ND-1000, Thermo Fisher Scientific, Waltham, MA, United States). The RNA samples were reverse-transcribed to cDNA with random primers using the RevertAid™ H Minus First Strand cDNA Synthesis kit (Life Technologies).

For both the DNA and cDNA, the V1–V3 region of the bacterial 16S rRNA genes were amplified using bacteria specific primers 27F AGRGTTTGATCMTGGCTCAG and 519R GTNTTACNGCGGCKGCTG with a single step and 28 cycles of PCR using the HotStarTaq Plus Master Mix Kit (Qiagen, Valencia, CA, United States). Following the PCR, all the amplicon products from the different samples were mixed in equal concentrations and purified using Agencourt Ampure beads (Agencourt Bioscience Corporation, MA, United States). Purified PCR products were used to prepare a DNA library by following the Illumina TruSeq DNA library preparation protocol. All samples were sequenced on the same Miseq Illumina sequencer run (Illumina, San Diego, CA, United States) using Miseq reagent kit V3 (Illumina) producing 2 × 300-bp long reads. PCR and sequencing were conducted in a commercial laboratory (MR DNA, Shallowater, TX, United States). All sequences were deposited in GenBank under SRA accession number SRP156494.

### Sequence Analysis

All the reads that had a mismatch with the 16S rRNA primers, contained ambiguous nucleotides (N) or were <300 bp long beyond the forward primer were removed. In addition, a stringent quality trimming criteria was applied to remove reads that had ≥10% of bases with Phred values <27. This procedure is recommended to ensure that when clustering at 97% or more, the influence of erroneous reads is minimized (Huse et al., 2010; Kunin et al., 2010). The sequences were then de-replicated and clustered at a 97% threshold using UCLUST (Edgar, 2010) for *de novo* operational taxonomic unit (OTU) picking. Representative sequences were classified against the SILVA v.128 database (Quast et al., 2013). Putative chimeric sequences were identified as sequences having a best Blast alignment <90% of the trimmed read length to the reference database, >90% sequence identity to the best Blast match and OTU size ≤2. Sequence analysis were performed with the PyroTagger pipeline (Kunin and Hugenholtz, 2010).

### Statistics

To compare the bacterial communities in the diversity analysis, all the samples were randomly re-sampled to match the size of the sample containing the fewest sequences (*n* = 8855). Possible differences in bacterial community composition were assessed by correspondence analysis (CA) with the vegan package in R (Oksanen et al., 2013) and significant differences were tested with ANOSIM. Possible differences between DNA and RNA community composition were measured by comparing the DNA versus the RNA samples scores along the first CA axes. The linear increase in diversity was tested using ordinary least squares regression (OLS) models and the statistical significance of models described with F statistics in R. The Bray Curtis index was computed between all samples with the vegan package in R.

## Results and Discussion

**FIGURE 1:**
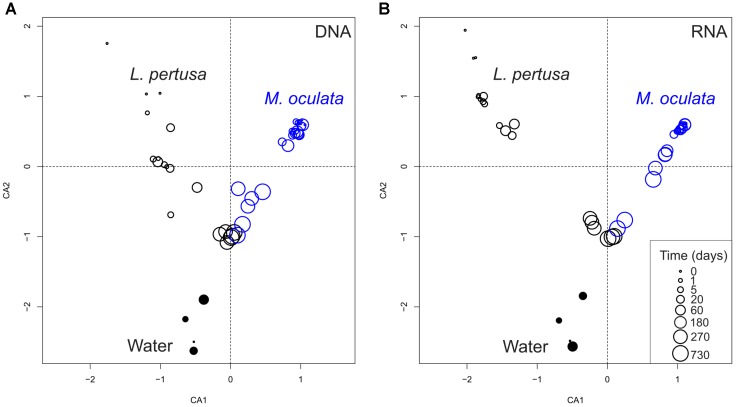
Correspondence analysis of bacterial communities based on 16S rDNA (DNA) **(A)** and 16S rRNA (RNA) **(B)**. *L. pertusa* communities are marked as black open circles, *M. oculata* as blue open circles, and water as filled circles. The size of the circles corresponds to the time in captivity.

Our experiment shows that the temporal dynamics of the coral microbiome, for both the active fraction (RNA) and the standing stock of bacteria (DNA), differed between *L. pertusa* and *M. oculata* (Figure 1). *M. oculata* maintained its associated community for the first 6 months of captivity, and the pattern was similar for both the active fraction and the standing stock. Inversely, the bacterial communities associated with *L. pertusa* changed rapidly and differences between wild and captive corals were seen already after 1 day of rearing (Figure 1). We also observed that for both the RNA and DNA fractions, *L. pertusa* and *M. oculata* had different bacterial community composition, and that the coral communities were different from the water communities (Supplementary Table 1). Interestingly, the community composition of *L. pertusa* and *M. oculata* converged with captivity time, and after 730 days there was no significant difference in community composition between the two coral species for both the DNA and RNA fractions (ANOSIM, *R* = 0.33 and 0.19 and *p* = 0.1 for the DNA and RNA fraction, respectively) (Figure 1).

Our results cast a new light on the effect of captivity on the coral holobiont. We show that captivity can rapidly impact the associated bacterial communities of one coral species but not of the other one at a 6 months’ time scale. After 1 year of captivity, however, both coral microbiomes had changed and lost their species-specific characteristics. The observation of different dynamics is interesting because captivity has been thought to always transform bacterial communities, as seen earlier for skeletal and mucus associated bacteria of *L. pertusa* (Schöttner et al., 2009) or during long term rearing of the deep-sea coral *Eguchipsammia fistula* (Röthig et al., 2017). It should, however, be noted that the differences observed in earlier studies may have been caused, in part, by sampling at different seasons (Schöttner et al., 2009), or by the use of different fixation methods (Schöttner et al., 2009). A few studies on tropical corals have also showed changes with captivity for *Siderastrea siderea* (Pratte et al., 2015), and *Fungia granulosa*, but on two samples only for the later (Kooperman et al., 2007).

**FIGURE 2:**
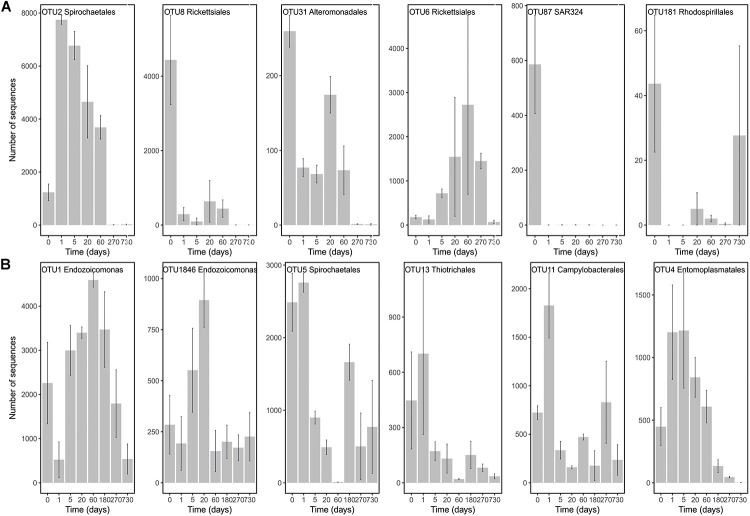
Number of sequences in the RNA fraction of the most abundant OTUs present in *L. pertusa* **(A)** and *M. oculata* **(B)** at different times of captivity. Detailed taxonomic affiliation for each OTUs is given in Supplementary Table 1.

**FIGURE 3:**
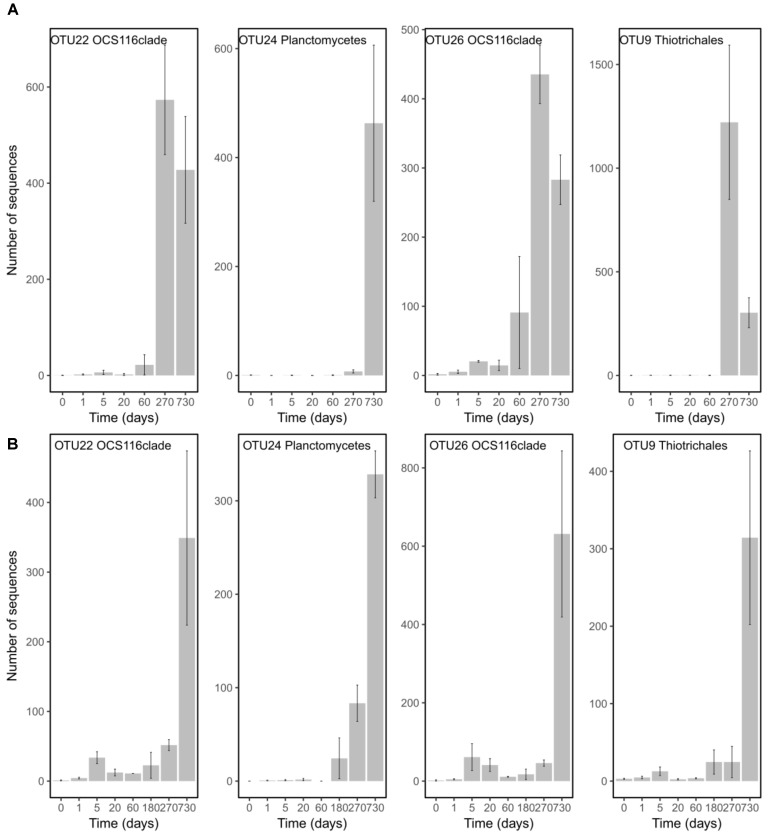
Number of sequences in the RNA fraction of OTUs characterizing time 270 and 730 and that were present in both species. Abundances are given for *L. pertusa* **(A)** and *M. oculata* **(B)** at different time of captivity. Detailed taxonomic affiliation for each OTUs is given in Supplementary Table 2.

**FIGURE 4:**
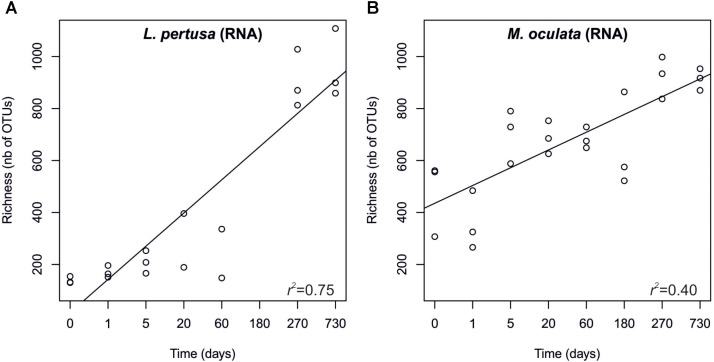
Richness of the RNA fraction of the bacterial communities in *L. pertusa* **(A)** and *M. oculata* **(B)**. Sampling time corresponds to the number of days during which the corals were maintained in captivity.

In order to pinpoint the pattern lying behind the changes in community composition we further identified the main bacteria associated to both cold-water corals species (Figure 2 and Supplementary Table 1). The six most abundant species specific bacteria represented 76 and 75% of the sequences in *L. pertusa* and *M. oculata*, respectively. Our results show that different bacterial taxa had different patterns of responses at the OTU level (Figure 2). *Endozoicomonas*, a typical symbiont found in various marine hosts (Neave et al., 2016), has been shown to have a stable relationship with *M. oculata* (Meistertzheim et al., 2016). In the RNA fraction, the number of *Endozoicomonas* sequences dropped significantly after 1 day of *M. oculata* captivity, but increased again after 5 days and hold for 6 months (Figure 2). It suggests that collection and the subsequent change of environment had an immediate effect on the activity of *Endozoicomonas*. However, this bacterium seems to rapidly adapt to the conditions found in aquarium and to be able to thrive for the first 6 months of captivity. This observed resilience is in line with the recent report of persistent *Endozoicomonas* phylotypes under coral bleaching and colony mortality (Pogoreutz et al., 2018). Other *M. oculata* bacteria were not as successful and showed a sharp decline in their relative number of RNA sequences during captivity (Figure 2). For *L. pertusa*, we also identified species specific bacteria that had been found earlier associated to the coral species *in situ* (Figure 2 and Supplementary Table 1). Some of these bacteria showed a sharp decrease in their RNA sequence abundance, or even a disappearance after 1 day of captivity, while other increased in relative sequence abundance (Figure 2). We hypothesize that the loss of some bacterial taxa released ecological niches that were quickly occupied by other bacteria, the ones that showed an increase in sequence number. We also identified bacteria that were found only at the end of the experiment (Figure 3), and which we considered as signature for the aquaria effect on the microbiome. These bacteria had never been found earlier associated to hosts (Supplementary Table 1), and their representative sequences were similar to sequences from bacteria growing on sediment or mineral substrates in the sea. It suggests that opportunist bacteria colonized the coral surface during captivity. Our observation of a significant increase in bacterial community richness with time of captivity for *L. pertusa* and *M. oculata* (*F* = 50.25, *P* < 0.001 and *F* = 14.59, *P* < 0.001, respectively) (Figure 4), which was observed earlier for *L. pertusa* (Schöttner et al., 2009), confirms the appearance of additional coral associated bacterial species during captivity. It shows that corals lost their ability to strongly select specific bacteria, which in turn may reflect a poorer health status. The fact that corals held in captivity for 10 months showed similar growth rates as corals *in situ* (Lartaud et al., 2014) suggests, however, a limited impact of captivity. Captivity’s effect on coral health remains to be tested exhaustively.

**FIGURE 5:**
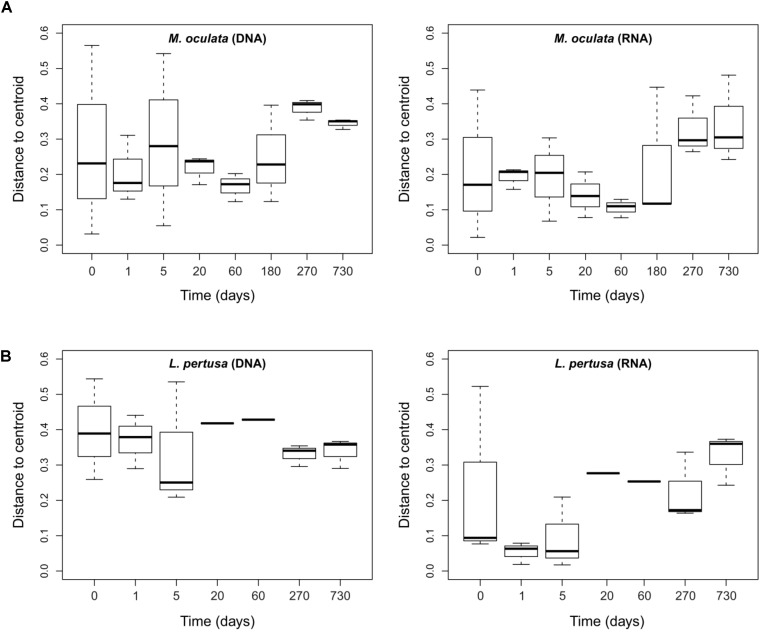
Dispersion of the bacterial communities’ beta-diversity at different times of captivity for the DNA and RNA fraction in *M. oculata* **(A)** and *L. pertusa* **(B)**. The dispersion is expressed as the distance to the centroid for each Bray Curtis values computed between replicates of a same category.

To further assess the effect of captivity on the corals’ microbiomes we measured beta diversity between samples at each sampling time. We observed that for the RNA fraction in *L. pertusa* the beta-diversity dispersion was low early in captivity, increased after 3 weeks and remained high for the rest of the experiment (Figure 5). For *M. oculata*, the beta-diversity dispersion was stable for the first 6 months of captivity and then increased (Figure 5). Such patterns of microbial community change can theoretically be used to understand how certain stressors affect host associated microorganisms (Zaneveld et al., 2017). In particular, the Anna Karenina Principle (AKP) predicts that certain stressors have stochastic rather than deterministic effects on community composition (Zaneveld et al., 2017). In the case of cold water corals, our results indicate that, if we consider capture and captivity as a stressor, the effect on *L. pertusa* was deterministic during the first week. The communities in the different polyps all changed toward a similar composition. Later, the effect became stochastic with communities having different composition in the different polyps. In *M. oculata*, the stabile beta diversity patterns during the first 6 months of captivity reflected the stabile community composition.

**FIGURE 6:**
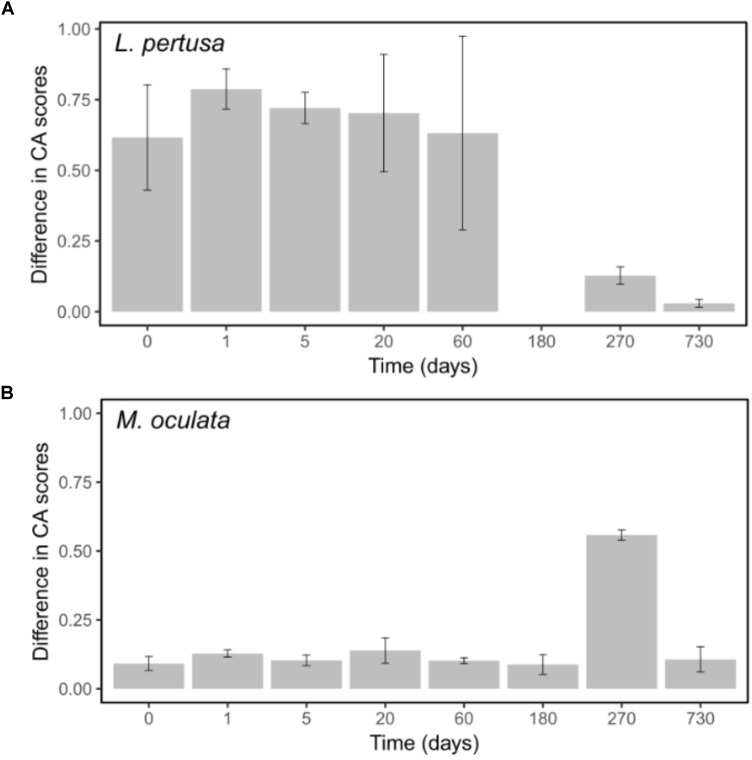
Differences between DNA and RNA sample scores on the first CA axes for *L. pertusa* **(A)** and *M. oculata* **(B)**. Bars represent the standard error of the mean.

The differences observed between *L. pertusa* and *M. oculata* confirm that they have species specific bacterial communities even though they share the same habitat (Hansson et al., 2009; Schöttner et al., 2012; Meistertzheim et al., 2016). The difference was hypothesized to correspond to different ecological strategies between the two species with a resilient consortium for *M. oculata* that fits the definition of holobiont, and more versatility for *L. pertusa*, which suggests an association that is more influenced by environmental conditions or nutritional status (Meistertzheim et al., 2016). Our results appear to validate that hypothesize. The difference seen between the RNA and DNA fraction for *L. pertusa* indicates that not all the bacteria that are present on the coral are active (Figure 6). It suggests the presence of opportunist bacteria, probably sticking to the coral, that are not active and thus probably without ecological relevance. Inversely, the similar DNA and RNA structure in *M. oculata* (Figure 6) may reflect a strong selection for a specific group of bacteria that is active, and the elimination of the less fit inactive organisms.

It should be noted that although the use of RNA has been shown to be a good indicator of the metabolic state for certain marine microbes (Salter et al., 2015), its use has been criticized (Blazewicz et al., 2013). However, in our study, the marked difference that we observed in DNA versus RNA communities between the *L. pertusa* and *M. oculata* (Figure 6) is a strong indication that the two species have very different types of relationship to their associated microbiome. The fact that the *M. oculata* microbiome is maintained during several months in aquaria argues for a stable relationship between the bacteria and the host. Inversely, the *L. pertusa* microbiome appears to change with changes in its surrounding environment. It remains to be shown if the plasticity of the *L. pertusa* holobiont can give competitive advantage or better fitness in a changing environment.

## Conclusion

We show that for some coral species, maintenance in captivity does not alter significantly the associated bacterial communities for at least 6 months. For others, the microbiome composition and activity change very quickly, as fast as within a day. Our results also highlight that different coral species have different types of associations to bacterial communities, which can be coined as “unfaithful” versus “loyal.” It demonstrates that a good knowledge of the coral microbiome and an understanding of the ecological strategy of the holobiont is needed before designing aquarium experiments. The choice of the species to study and the duration of the experiment is crucial. Finally, we also reveal the notable resilience under aquarium conditions of the *Endozoicomonas* bacteria known as key symbionts in many marine hosts.

## Author Contributions

PG and FL designed the study. PG, FL, LC, A-LM, and EP conducted the study, analyzed data, and wrote the paper.

## Conflict of Interest Statement

The authors declare that the research was conducted in the absence of any commercial or financial relationships that could be construed as a potential conflict of interest.
